# Further studies on the morphology of three species of *Rhopalopsole* (Plecoptera, Leuctridae)

**DOI:** 10.3897/BDJ.13.e154980

**Published:** 2025-05-23

**Authors:** Xiao Yang, Yu-Zhou Du

**Affiliations:** 1 College of Plant Protection & Institute of Applied Entomology, Yangzhou University, Yangzhou 225009, China College of Plant Protection & Institute of Applied Entomology, Yangzhou University Yangzhou 225009 China; 2 Joint International Research Laboratory of Agriculture and Agri-Product Safety, the Ministry of Education, Yangzhou University, Yangzhou 225009, China Joint International Research Laboratory of Agriculture and Agri-Product Safety, the Ministry of Education, Yangzhou University Yangzhou 225009 China

**Keywords:** Plecoptera, *Rhopalopsole*, China, re-description

## Abstract

**Background:**

*Rhopalopsole* Klapálek, 1912, is a species-rich genus in the family Leuctridae, with more than 80 valid species known from the Oriental and eastern Palaearctic Regions.

**New information:**

We recently examined specimens of *Rhopalopsole* Klapálek, 1912 from Hubei and Guizhou, China and provided a supplementary description of *Rhopalopsoleampulla* Du & Qian, 2011, *Rhopalopsoleexiguspina* Du & Qian, 2011 and *Rhopalopsolememorabilis* Qian & Du, 2014. Additionally, we provide new images of these species to facilitate identification.

## Introduction

Currently, more than 70 species of *Rhopalopsole* Klapálek, 1912 have been widely recorded in southern China (DeWalt et al. 2024). Amongst these, *R.ampulla* Du & Qian, 2011, *R.exiguspina* Du & Qian, 2011 and *R.memorabilis* Qian & Du, 2014 are not assigned to any group according to Sivec et al. (2008). A detailed diagnosis was provided at the time of publication to distinguish these species. However, subsequent reports on the morphological diversity of *Rhopalopsole* (Yang et al. 2022, Yang and Du 2022, Huo et al. 2023) suggest that the differences between these species are minimal, indicating that their taxonomic status requires further evaluation. The type specimens of these three *Rhopalopsole* species were re-examined and the validity of the original features were reassessed in the style of Qian and Du (2011) and Qian et al. (2014). As some characters showed variation, we amend the original descriptions and provide new colour images of each species.

## Materials and methods

Specimens were collected by hand and preserved in 75% ethanol. Morphological details were examined with a Leica MZAPO microscope. Colour illustrations were taken with a KEYENCE VHX-5000. All specimens used in this study are deposited in the Insect Collection of Yangzhou University (ICYZU), Jiangsu Province, China. The morphological terminology follows that of Sivec et al. (2008).

## Taxon treatments

### Rhopalopsole ampulla

Du & Qian, 2011

17781D0A-1643-5862-9BA4-509AA9A6EEBB

#### Materials

- **Type status:** Holotype. **Occurrence:** occurrenceID: 74C14315-0843-5241-A949-FEA0B9D76F50; **Taxon:** scientificName: *Rhopalopsoleampulla*; order: Plecoptera; family: Leuctridae; genus: Rhopalopsole ; specificEpithet: *ampulla*; **Location:** country: China; stateProvince: Guizhou; county: Yanhe; locality: Shaba; verbatimElevation: 903 m; verbatimLatitude: 28.364549N; verbatimLongitude: 108.379991E
- **Type status:** Paratype. **Occurrence:** occurrenceID: 47DB17F6-BA99-5514-AFD8-44A1CB5858A6; **Taxon:** scientificName: *Rhopalopsoleampulla*; order: Plecoptera; family: Leuctridae; genus: Rhopalopsole ; specificEpithet: *ampulla*; **Location:** country: China; stateProvince: Guizhou; county: Yanhe; locality: Shaba; verbatimElevation: 903 m; verbatimLatitude: 28.364549N; verbatimLongitude: 108.379991E
- **Type status:** Paratype. **Occurrence:** occurrenceID: 89A5D3FF-BE29-516A-BD13-648630C9D849; **Taxon:** scientificName: *Rhopalopsoleampulla*; order: Plecoptera; family: Leuctridae; genus: Rhopalopsole ; specificEpithet: *ampulla*; **Location:** country: China; stateProvince: Guizhou; county: Yanhe; locality: Shaba; verbatimElevation: 903 m; verbatimLatitude: 28.364549N; verbatimLongitude: 108.379991E
- **Type status:** Paratype. **Occurrence:** occurrenceID: 1079CCEC-C33C-53F3-B407-7C8A17244304; **Taxon:** scientificName: *Rhopalopsoleampulla*; order: Plecoptera; family: Leuctridae; genus: Rhopalopsole ; specificEpithet: *ampulla*; **Location:** country: China; stateProvince: Guizhou; county: Yanhe; locality: Shaba; verbatimElevation: 903 m; verbatimLatitude: 28.364549N; verbatimLongitude: 108.379991E

#### Re-description

**Figure 2.**
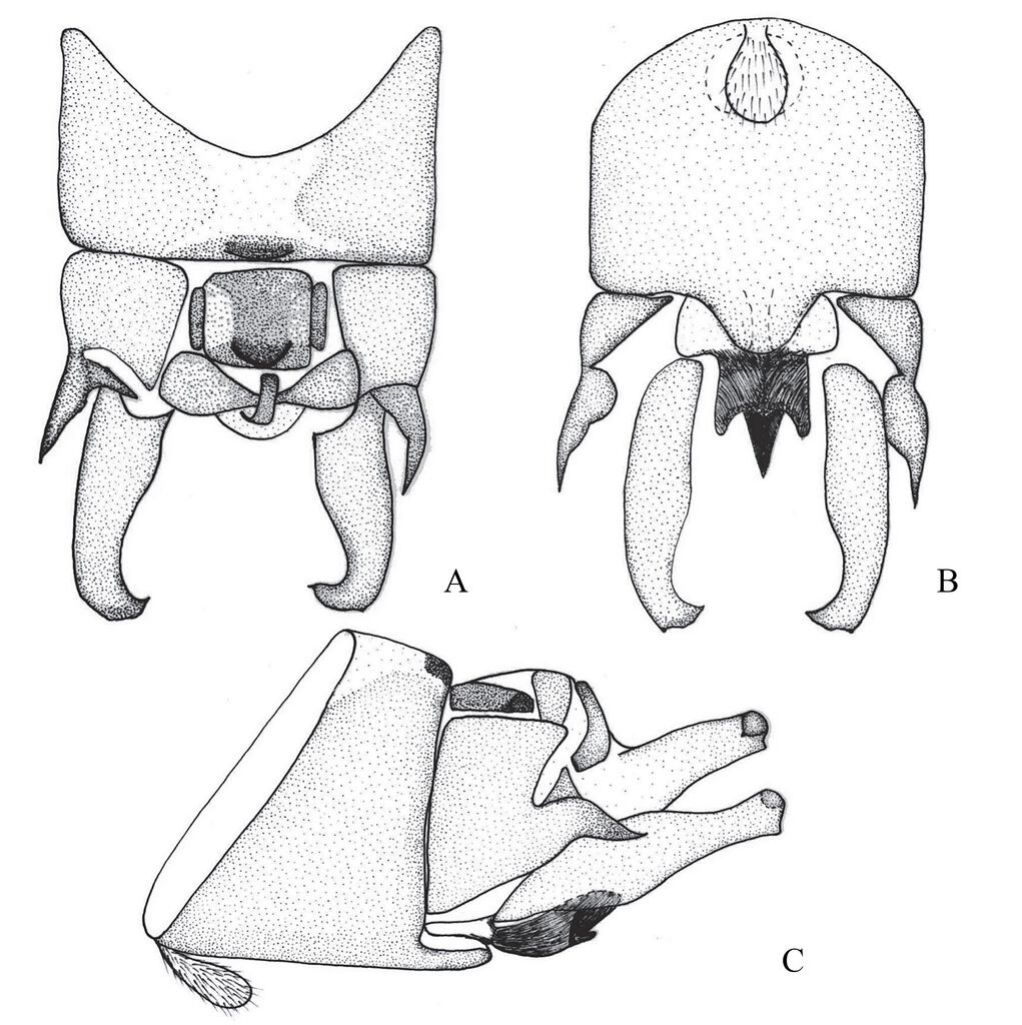
*Rhopalopsoleampulla* Du & Qian, 2011**. A** Male terminalia, dorsal view; **B** Male terminalia, ventral view; **C** Male terminalia, lateral view. (Cited from Qian and Du (2011)).

**Figure 3.**
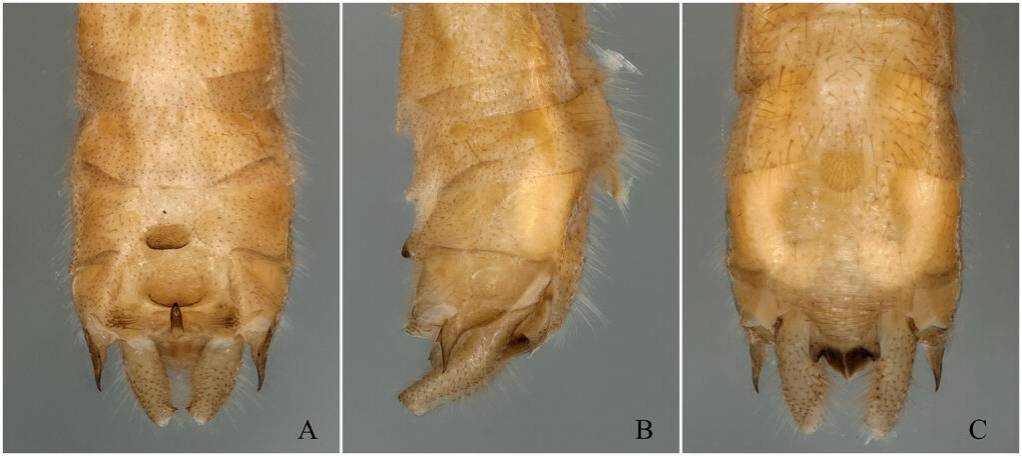
*Rhopalopsoleampulla* Du & Qian, 2011, holotype. **A** Male terminalia, dorsal view; **B** Male terminalia, lateral view; **C** Male terminalia, ventral view.

**Figure 4.**
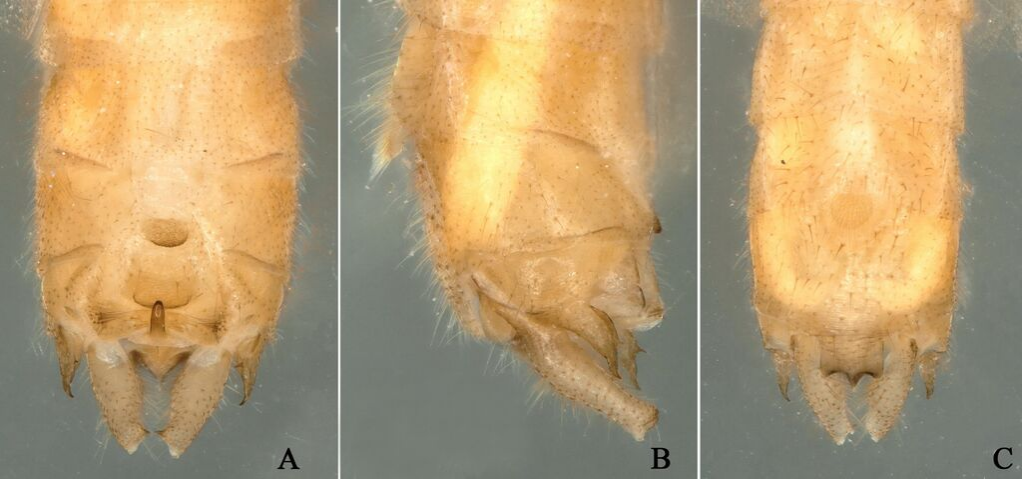
*Rhopalopsoleampulla* Du & Qian, 2011, paratype a. **A** Male terminalia, dorsal view; **B** Male terminalia, lateral view; **C** Male terminalia, ventral view.

**Figure 5.**
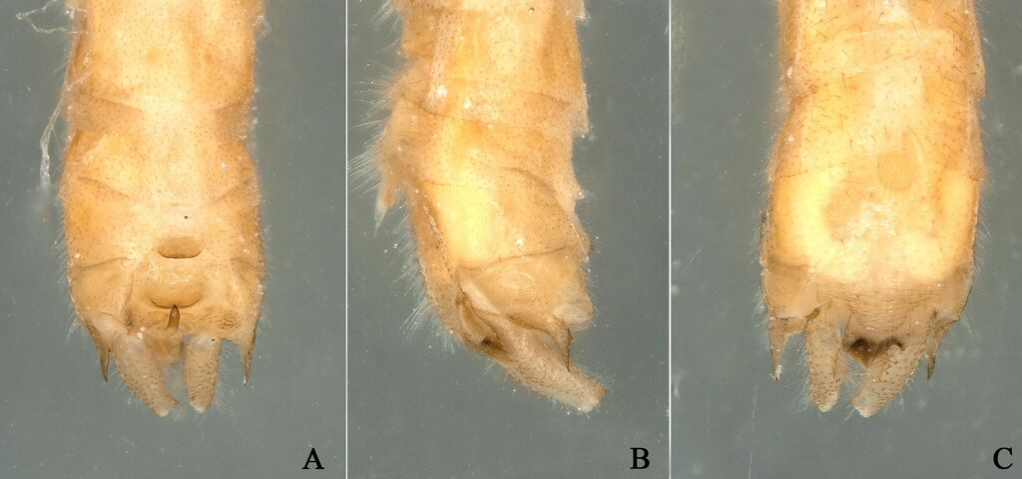
*Rhopalopsoleampulla* Du & Qian, 2011, paratype b. **A** Male terminalia, dorsal view; **B** Male terminalia, lateral view; **C** Male terminalia, ventral view.

**Figure 6.**
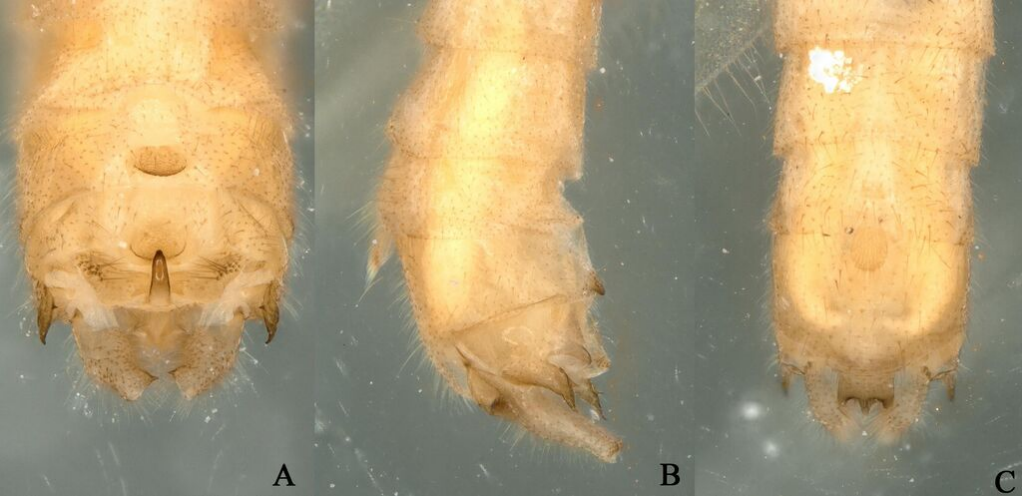
*Rhopalopsoleampulla* Du & Qian, 2011, paratype c. **A** Male terminalia, dorsal view; **B** Male terminalia, lateral view; **C** Male terminalia, ventral view.

Brown and dark brown. Head brown or dark brown, wider than prothorax, hind ocelli much closer to eyes than to each other, antennae and palpi brown. Prothorax dark brown, quadrate, longer than wide, all angles rounded and some black irregular stripes on it. Legs light brown. Wings hyaline and veins light brown. Forewing length 8 mm, body length 8.5 mm. Tergum 9 sclerotised with a large, medial membranous area, a sclerotised, semicircular process present posteromedially. Sternum 9 with a subgenital plate wider than long and rounded apically, basally with a tongue-like vesicle bearing dense hairs. Tergum 10 with one large broad median mid-anterior sclerite; mid-posterior more sclerotised and protrusive; one pair of transverse sclerite weakly sclerotised. Lateral processes each strongly sclerotised, spine-like rather than thick basally, narrowed apically and downward in lateral aspect. Epiproct curved forward, thick and blunt apically. Subanal lobe strongly sclerotised at base, trident-like apically in ventral aspect and membranous at its apex. Cerci long and cylindrical, ampulla-like, thick basally and thin apically, each with a tiny spine (Figs 1a, 2, 3, 4, 5, 6).

### Rhopalopsole exiguspina

Du & Qian, 2011

5D4AD404-E873-569B-9F11-EC0C9FF41D98

#### Materials

- **Type status:** Holotype. **Occurrence:** occurrenceID: BD041270-3DD4-5851-9734-D20488DAE7B5; **Taxon:** scientificName: *Rhopalopsoleexiguspina*; order: Plecoptera; family: Leuctridae; genus: Rhopalopsole ; specificEpithet: *exiguspina*; **Location:** country: China; stateProvince: Guizhou; county: Yanhe; locality: Shaba; verbatimElevation: 903 m; verbatimLatitude: 28.364549N; verbatimLongitude: 108.379991E

#### Re-description

**Figure 7.**
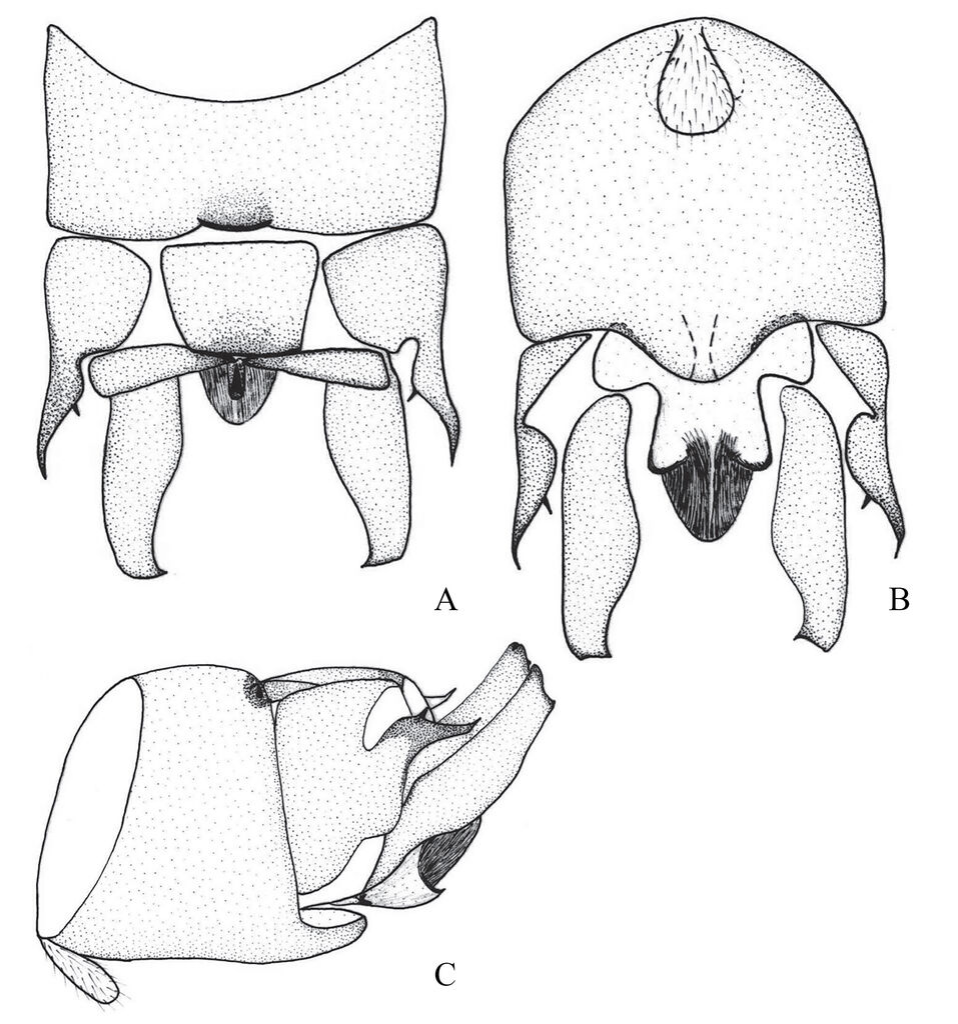
*Rhopalopsoleexiguspina* Du & Qian, 2011. **A** Male terminalia, dorsal view; **B** Male terminalia, ventral view; **C** Male terminalia, lateral view. (Cited from Qian and Du (2011)).

**Figure 8.**
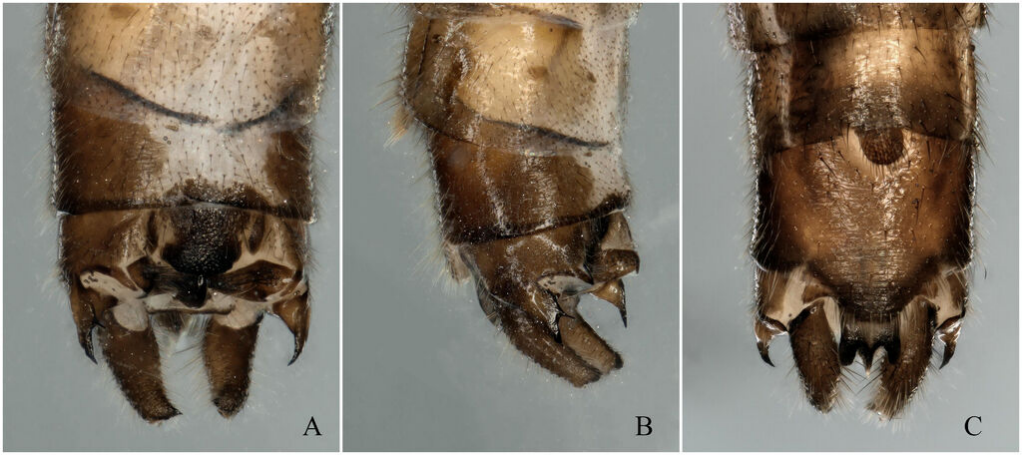
*Rhopalopsoleexiguspina* Du & Qian, 2011, holotype. **A** Male terminalia, dorsal view; **B** Male terminalia, lateral view; **C** Male terminalia, ventral view.

Head brown or light brown, wider than prothorax, hind ocelli much closer to the eyes than to each other, antennae and palpi yellowish-brown. Prothorax light brown, subquadrate, all angles somewhat rounded with some black irregular stripes on it. Legs light brown. Wings hyaline and veins light brown. Forewing length 6.0 mm, body length 6.5 mm. Tergum 9 sclerotised, with a X-shape central membranous area. Sternum 9 basally with a tongue-like vesicle bearing dense hairs, apically with a subgenital plate wider than long and rounded apically. Tergum 10 with strongly sclerotised lateral process beak-like somewhat acute and curving inwards apically and a small spine at the middle of lateral process in dorsal view, thick basally and slightly curved upwards apically in lateral view. Mid-anterior sclerite sclerotised, posterior margin more sclerotised; one pair of transverse triangle sclerite weakly sclerotised. Epiproct with a simple curved process, erect hook-like apical portion curved inwards. Subanal lobe strongly sclerotised at base, trident-like apically in ventral aspect and membranous at its apex (Figs 1b, 7, 8).

### Rhopalopsole memorabilis

Qian & Du, 2014

5B950BF9-CF80-52ED-87D3-F7E92ACD1CE3

#### Materials

- **Type status:** Holotype. **Occurrence:** occurrenceID: 67F46ED9-429C-5DC6-99D2-39B3EAA6C477; **Taxon:** scientificName: *Rhopalopsolememorabilis*; order: Plecoptera; family: Leuctridae; genus: Rhopalopsole; specificEpithet: *memorabilis*; **Location:** country: China; stateProvince: Hubei; county: Shennongjia; locality: Shimo; verbatimElevation: 800-900 m; verbatimLatitude: 31.486807 N; verbatimLongitude: 110.312168 E
- **Type status:** Other material. **Occurrence:** occurrenceID: B89D7023-B9E3-5CBC-8F78-33DA92319540; **Taxon:** scientificName: *Rhopalopsolememorabilis*; order: Plecoptera; family: Leuctridae; genus: Rhopalopsole; specificEpithet: *memorabilis*; **Location:** country: China; stateProvince: Hubei; county: Shennongjia; locality: muyu; verbatimElevation: 1200-1220 m; verbatimLatitude: 31.473053 N; verbatimLongitude: 110.38959 E

#### Re-description

**Figure 9.**
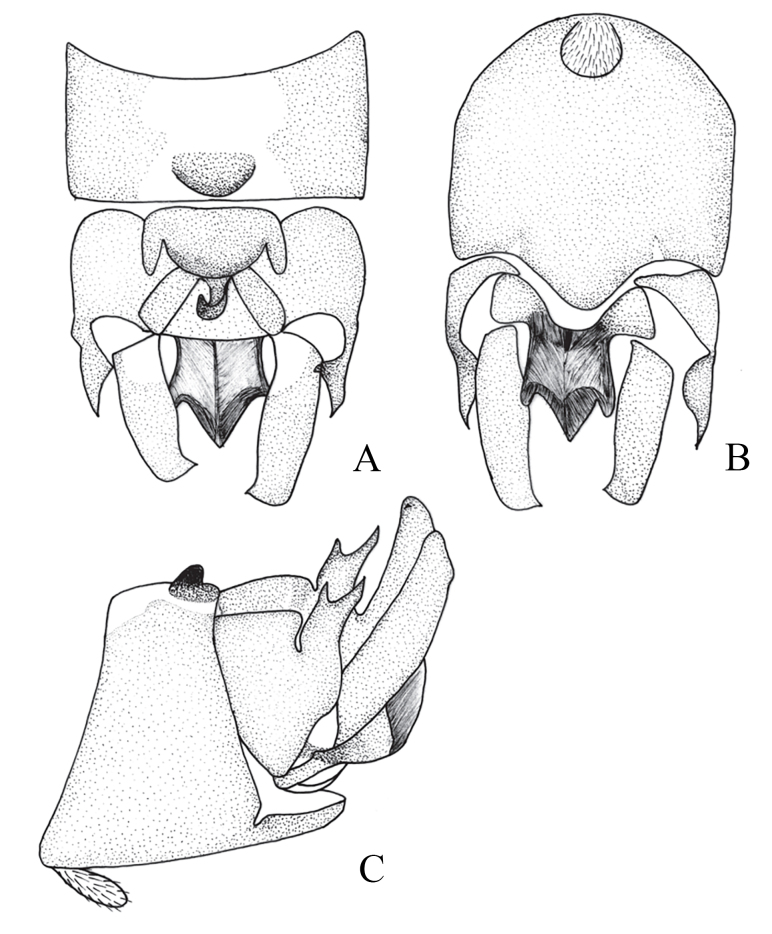
*Rhopalopsolememorabilis* Qian & Du, 2014. **A** Male terminalia, dorsal view; **B** Male terminalia, ventral view; **C** Male terminalia, lateral view. (Cited from Qian et al. (2014)).

**Figure 10.**
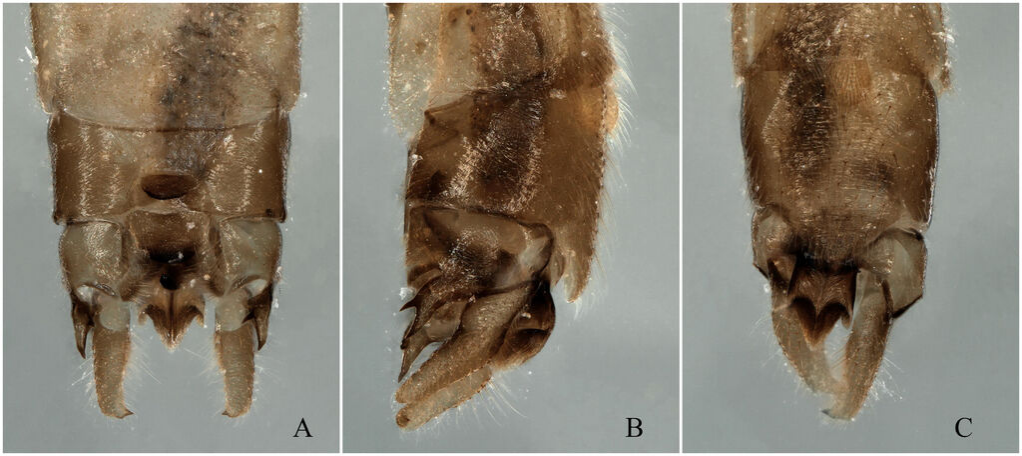
*Rhopalopsolememorabilis* Qian & Du, 2014. holotype **A** Male terminalia, dorsal view; **B** Male terminalia, lateral view; **C** Male terminalia, ventral view.

**Figure 11.**
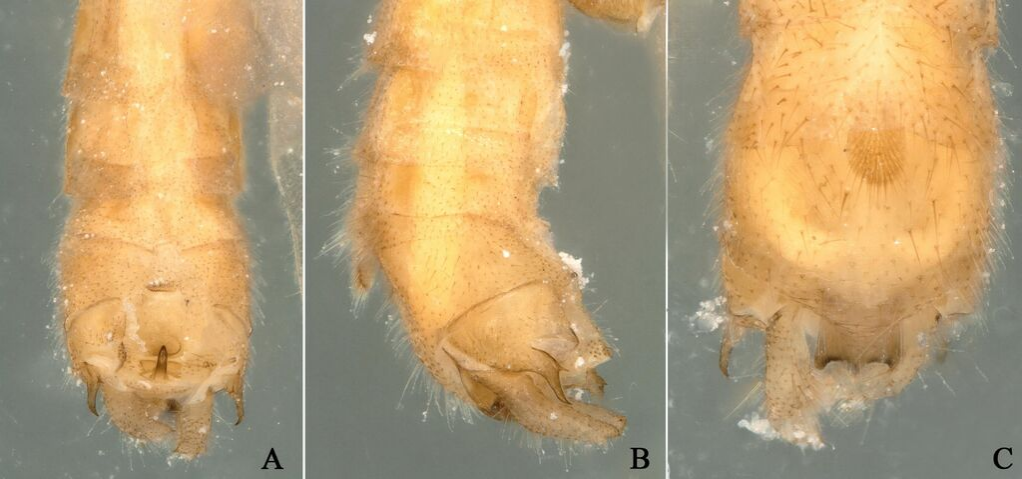
*Rhopalopsolememorabilis* Qian & Du, 2014. other material **A** Male terminalia, dorsal view; **B** Male terminalia, lateral view; **C** Male terminalia, ventral view.

Body length 7.0 mm, forewing length 8.0 mm. Tergum 9 sclerotised with a large, medial membranous area, a sclerotised, semicircular process present posteromedially, an obvious ridge across the middle part of the process. Sternum 9 as wide as long, forming a rounded projection apically that is no wider than the subanal lobe bases, the ventral lamella somewhat broadly circular and densely hairy. Sclerotised lateral processes of tergum 10 acutely bifurcate apically in lateral view. Mid-anterior process sclerotised, distinctly wider than long and bearing two short obtuse lateral processes. Posterior transverse sclerites rectangular with posterior angles somewhat rounded. Epiproct thick with erect, hook-like portion curved dorsally. Subanal lobe strongly sclerotised at base and margin, membranous distally, overall appearance is trident-like, lateral arms short and less massive than the medial section. Cerci long and cylindrical, in lateral view, gently curved dorsally, a small, subapical, medially-directed spine present (Figs 1c, 9, 10, 11).

## Discussion

Qian and Du (2011) described *R.ampulla* as having a sclerotised tergite 9, with a large central membranous area and strongly sclerotised mid-posterior margin (Fig. 2). After examining the holotype of *R.exiguspina*, we found that *R.ampulla* also has the same distinct transverse ridge medially in lateral view (Figs 3, 8). In *R.ampulla*, the lateral lobe of tergum 10 is very small, differentiating it from *R.exiguspina*.

Qian and Du (2011) stated that the subanal lobes of *R.exiguspina* are rounded apically and each possesses a small spine at the middle of lateral process (Fig. 7), whereas those of *R.ampulla* are strongly sclerotised and trident-like apically in ventral view (Fig. 3). However, perhaps due to the limitations of the equipment at that time and the influence of the drawing angle, upon re-examining the type specimen, we found that the inferior anal lobe of *R.exiguspina* is not rounded apically, but trident-like apically in ventral view (Fig. 8). Both *R.memorabilis* and *R.ampulla* have a significant semicircular process posteromedially on tergum 9 that bears a distinct transverse ridge medially, whereas *R.exiguspina* lacks this posteromedial structure.

Qian et al. (2014) stated that *R.memorabilis* differs from *R.ampulla* in that *R.memorabilis* has a large semicircular process posteromedially on tergum 9, which bears a distinct transverse ridge medially; the lateral lobe of tergum 10 is bifurcate apically and the cerci exhibit a small, sessile spine subapically (Figs 9, 10). However, upon examining the type specimen, we found that *R.ampulla* also has the same distinct transverse ridge medially (Fig. 3).

The subanal lobes of all three species are very similar, with the greatest difference being the shape of the sclerotised lateral processes of tergum 10.

## Supplementary Material

XML Treatment for Rhopalopsole ampulla

XML Treatment for Rhopalopsole exiguspina

XML Treatment for Rhopalopsole memorabilis

## Figures and Tables

**Figure 1a.**
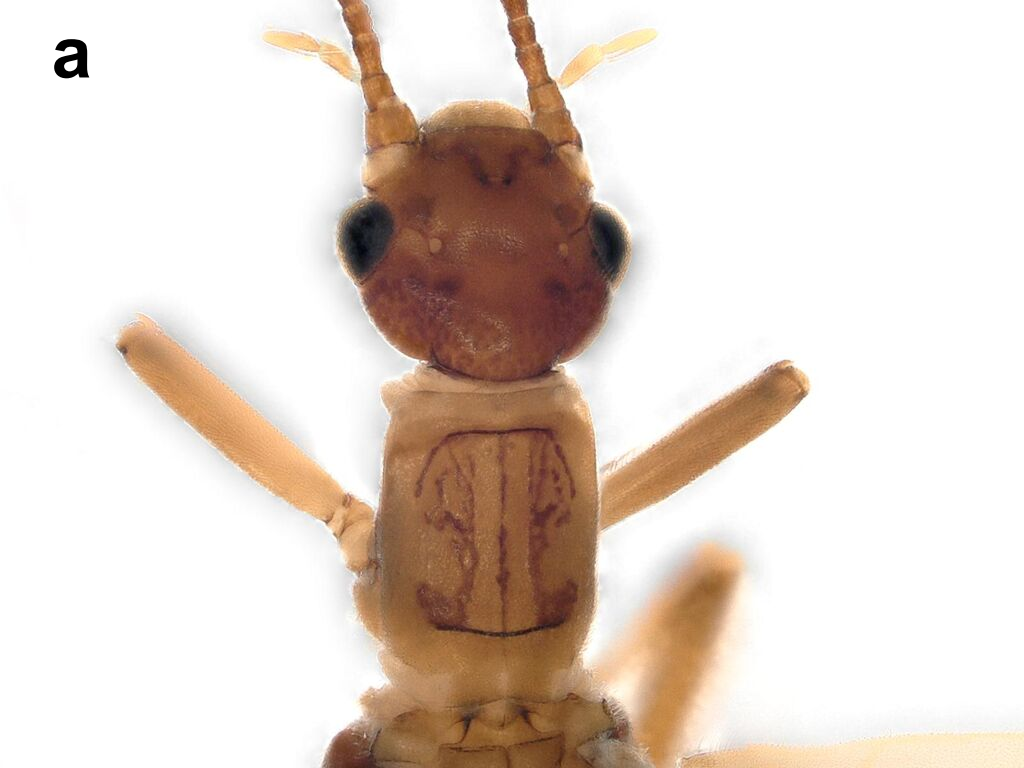


**Figure 1b.**
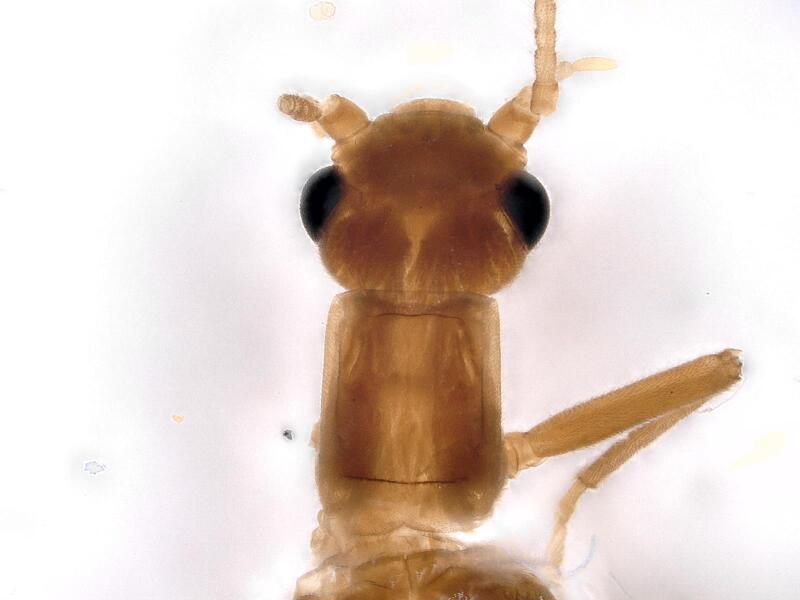


**Figure 1c.**
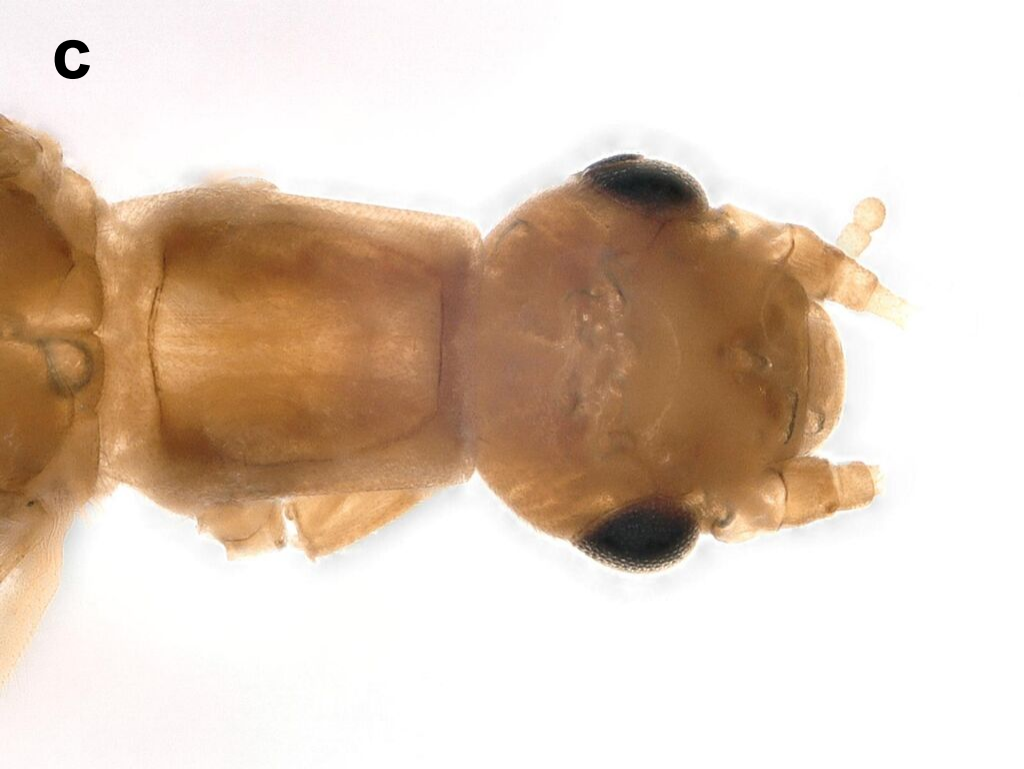


## References

[B12678473] DeWalt R. E., Hopkins H., Neu-Becker U., Stueber G. Plecoptera Species File. https://plecoptera.speciesfile.org.

[B12678481] Huo Q. B., Zhao M. Y., Du Y. Z, Muranyi D., Zhu B. Q., Yu L. (2023). The intraspecific morphological variability of *Styloperla* Wu, 1935 (Plecoptera: Styloperlidae). Zootaxa.

[B12678492] Qian Y. H., Du Y. Z. (2011). Two new species of genus *Rhopalopsole* (Insecta, Plecoptera, Leuctridae) form China. ZooKeys.

[B12678501] Qian Y. H., Li Y. H., Du Y. Z. (2014). A study of Leuctridae (Insecta: Plecoptera) from Shennongjia, Hubei Province, China.. Florida Entomologist.

[B12678520] Sivec I., Harper P. P., Shimizu T. (2008). Contribution to the study of the Oriental genus *Rhopalopsole* (Plecoptera: Leuctridae). Scopolia.

[B12678529] Yang Y. B., Du Y. Z. (2022). Two new synonyms of *Paraleuctraorientalis* (Chu, 1928) (Plecoptera: Leuctridae) based on morphological and molecular data, with notes on *Paraleuctracervicornis* Du and Qian, 2012. Insects.

[B12678538] Yang Y. B., Du Y. Z., Zhu B. Q. (2022). A new species of *Rhopalopsole* Klapálek, 1912 with preliminary female identifications in this genus. Zootaxa.

